# Novel approach to exploring protease activity and targets in HIV-associated obstructive lung disease using combined proteomic-peptidomic analysis

**DOI:** 10.1186/s12931-024-02933-9

**Published:** 2024-09-10

**Authors:** Sarah Samorodnitsky, Monica Kruk, Eric F. Lock, Ken M. Kunisaki, Alison Morris, Janice M. Leung, Danielle Weise, Subina Mehta, Laurie L. Parker, Pratik D. Jagtap, Timothy J. Griffin, Chris H. Wendt

**Affiliations:** 1 Biostatistics Division, School of Public Health, University of Minnesota, Minneapolis, MN USA; 2 Department of Medicine, University of Minnesota, Minneapolis, MN USA; 3 Department of Medicine, Minneapolis VA Health Care System, Minneapolis, MN USA; 4 Department of Medicine, University of Pittsburgh School of Medicine, Pittsburgh, PA USA; 5 Department of Medicine, University of British Columbia, Vancouver, Canada; 6 Department of Biochemistry, Molecular Biology and Biophysics, University of Minnesota, Minneapolis, MN USA

## Abstract

**Background:**

Obstructive lung disease (OLD) is increasingly prevalent among persons living with HIV (PLWH). However, the role of proteases in HIV-associated OLD remains unclear.

**Methods:**

We combined proteomics and peptidomics to comprehensively characterize protease activities. We combined mass spectrometry (MS) analysis on bronchoalveolar lavage fluid (BALF) peptides and proteins from PLWH with OLD (n = 25) and without OLD (n = 26) with a targeted Somascan aptamer-based proteomic approach to quantify individual proteases and assess their correlation with lung function. Endogenous peptidomics mapped peptides to native proteins to identify substrates of protease activity. Using the MEROPS database, we identified candidate proteases linked to peptide generation based on binding site affinities which were assessed via z-scores. We used t-tests to compare average forced expiratory volume in 1 s per predicted value (FEV1pp) between samples with and without detection of each cleaved protein and adjusted for multiple comparisons by controlling the false discovery rate (FDR).

**Findings:**

We identified 101 proteases, of which 95 had functional network associations and 22 correlated with FEV1pp. These included cathepsins, metalloproteinases (MMP), caspases and neutrophil elastase. We discovered 31 proteins subject to proteolytic cleavage that associate with FEV1pp, with the top pathways involved in small ubiquitin-like modifier mediated modification (SUMOylation). Proteases linked to protein cleavage included neutrophil elastase, granzyme, and cathepsin D.

**Interpretations:**

In HIV-associated OLD, a significant number of proteases are up-regulated, many of which are involved in protein degradation. These proteases degrade proteins involved in cell cycle and protein stability, thereby disrupting critical biological functions.

**Supplementary Information:**

The online version contains supplementary material available at 10.1186/s12931-024-02933-9.

## Introduction

The use of highly active antiretroviral therapy (ART) has significantly reduced morbidity and mortality among those living with HIV. However, as life expectancy has increased, there has been a rise in co-morbidities, including obstructive lung disease (OLD) [1–8]. Persons living with HIV (PLWH) are at increased risk of accelerated lung function decline and developing OLD, even after adjusting for smoking [9, 10]. The Global Initiative for Obstructive Lung Disease (GOLD) 2024 report now recognizes HIV as a risk factor for COPD [11].

In non-HIV associated OLD, chronic inflammation and the activation of proteases play a crucial role in its pathogenesis. The severity of airflow obstruction often corresponds to the degree of inflammation in the lung and airways. In PLWH, various factors have been implicated in the development of OLD, such as epigentic aging, chronic systemic inflammation, innate immune activation, and abnormal immune function related to HIV [12, 13]. Furthermore, in PLWH who smoke and have emphysema, there is an upregulation of matrix metalloprotineases (MMP−1, −7, −9 and −12) compared to HIV-negative individuals, underscoring the potentially significant role of proteases in OLD pathogenesis in PLWH [14].

Numerous proteases contribute to lung disease, falling into three general categories that include serine proteases, cysteine proteases, and matrix metalloproteinases (MMP). The cellular sources of these proteases include inflammatory cells such as neutrophils and macrophages, as well as bronchial epithelial cells. While the impact of individual proteases on the lung extracellular matrix (ECM) has been well-documented in numerous studies, the extent to which other proteins are subject to proteolytic degradation and the physiological effects of this destruction remain relatively unknown. Previous studies have predominantly focused on individual proteases or their families in OLD, mainly limited to ECM targets. To better understand the role of proteases in HIV-associated OLD, we used complementary proteomic techniques combined with peptidomics to identify active proteases associated with OLD, comparing their activity in disease versus health and elucidating their specific targets.

## Methods

### Study population

PLWH who had undergone bronchoscopy were selected from the Pittsburgh and Vancouver Lung HIV Cohorts [15, 16]. This consisted of individuals (n = 25) with OLD as defined as the ratio of forced expiratory volume in 1-s/forced vital capacity (FEV1/FVC) < lower limit of normal. Those without OLD consisted of 26 individuals with HIV and normal lung function (defined as FEV1/FVC > lower limit of normal and FEV1 > 80% of predicted normal) matched on age (± 5 years), antiretroviral treatment use, and smoking status (current vs. non-smoker). Participants in the parent cohort studies provided informed consent for BALF collection and storage with approval by their respective Institutional Review Boards at Pittsburgh and Vancouver. At study enrollment, BALF was collected on fasting participants as previously described [15, 16]. Pulmonary function tests were performed within 3 months of collecting the samples. All data and samples were sent to the University of Minnesota were de-identified. The current study was reviewed in adherence to the Declaration of Helsinki and accepted by the University of Minnesota Institutional Review Board (Number 00003486).

### Protein processing and protease identification

BALF samples underwent centrifugation at the local collection sites to remove cells, and cell-free BALF samples were stored at -80 degrees Celsius prior to processing. The BALF was processed as previously described [17]. Briefly, the cell-free BALF samples were centrifuged twice to separate out the insoluble component of BALF from the soluble fraction. Endogenously produced peptides were collected from the soluble component of the supernatant via a 3 kDa MW cutoff filter. The soluble component of the supernatant was sent for SomaScan, analysis as previously reported, and MS analysis [18]. BALF samples from 21/25 with OLD and 24/26 with normal lung function had adequate protein amounts for tandem mass tagging (TMT, Thermo Fisher Scientific) and MS analysis. The insoluble BALF component was also processed for TMT labelling and liquid chromatography (LC) tandem mass spectrometry (MS/MS) analysis. Proteins were matched to UniProt IDs using Fragpipe. The combined proteins from SomaScan and MS were filtered to identify proteases and peptidases with known substrates based on the MEROPS database [19]. We utilized the STRING database to visualize protein networks [20].

### Peptide analysis and protease assignment

**Fig. 1:**
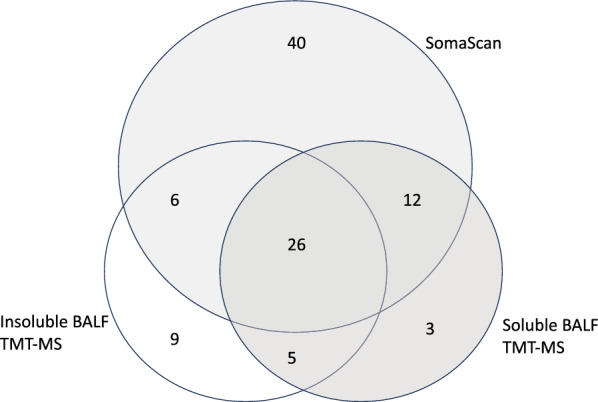
Venn diagram of proteases that correlate with FEV1pp from the various BALF proteomic analyses

The endogenous peptides isolated from the BALF underwent label-free identification and quantification by LC–MS that included delayed normalization and maximal peptide ratio extraction (MaxLFQ). A FASTA database was downloaded containing protein sequences of the entire human proteome (UniProt proteome sequence 2021-12-10, 101,014 protein sequences). The peptide tandem mass spectra (MS/MS) files were matched to the FASTA files using the Fragpipe software and were assigned to their native protein substrates [21–28]. Peptides matched with the Fragpipe software were quantified using the MaxLFQ method, and assigned cleavage sites. The cleavage sites were categorized by type of cleave based on cleave location and whether other similar peptides were detected, indicative of multiple cleavage events. The cleaves assigned were a result of exopeptidase or endopeptidase activity and mapped back to the original FASTA protein sequence with 4 residues before and after each cut, depending on location of the cut, based on starting residue and peptide length (Fig. 1S). The MEROPS catalog of preferred substrate patterns of cleavage was compared to our assigned cleavages from detected peptides. For each protease, a z-score was calculated for each cleave using z = (*x*-μ)/σ where *x* was the number of substrates in the MEROPS database with a given amino acid at a specific position, μ was the average number of substrates with any data for that cleave position, and σ for the standard deviation of the substrates for that cleave position. We treated the z-scores as a quantitative indicator for whether the peptide matches the protease’s target cleavage sequence. A higher z-score implied a higher likelihood that the protease cleaved a protein and yielded the corresponding peptide. We assigned cleaved proteins to proteases if the associated z-score was deemed an “outlier.” To define an outlier, we computed the z-score quartiles and interquartile range (IQR) within each protease. We defined an outlier as a peptide’s z-score exceeding the third quartile plus 1.5 times the interquartile range for that protease.

### Statistical analysis

All data underwent cleaning prior to performing statistical analysis (see Supplement). We sought to describe associations between the detected proteases along with the degraded proteins mapped from the endogenous peptides with measures of lung disease, defined as percent predicted forced expiratory volume in 1 s (FEV1pp).

### Proteases associated with lung function

We examined the overall association between protease abundance and FEV1pp using the combined SomaScan and two untargeted MS datasets from the soluble and insoluble components of BALF. For each identified protease, we calculated the correlation between the measured abundance and FEV1pp to the SomaScan and the two untargeted datasets. For the SomaScan dataset, we averaged the correlations across aptamers and proteins detected across datasets if multiple aptamers were present. We used the p-values from a Pearson correlation test to assess the strength of association between protease abundance and FEV1pp. We obtained an overall p-value for each protease by aggregating the individual p-values using Fisher’s combination method [29]. We controlled the false discovery rate (FDR) using the Benjamini–Hochberg correction [30]. We report on associations that were significant at the FDR < 0.05 level.

### Association between protein degradation and disease

For each protein assigned to an endogenous peptide, we dichotomized patients into two groups: one in which the degraded protein was detected and one in which it was not. A protein was “detected” if its corresponding MaxLFQ intensity was non-zero. Due to heavy missingness, we only considered proteins detected in at least five samples. We compared the average forced expiratory volume in 1-s (FEV1pp) between these two groups for each protein using a two-sample t-test. We controlled the Benjamini–Hochberg FDR [31]. For pathway analysis we used a less stringent FDR of below the 0.1 level using IMPaLa software to examine pathways reflected among the degraded proteins. [32]

## Results

### Study Participant Demographics

Table 1 summarizes the demographics of participants whose samples were used in the endogenous peptides analysis. The soluble and insoluble components of BALF TMT datasets differed by two samples from individuals with OLD and the SomaScan dataset differed by one sample from an individual with OLD, but overall showed similar demographic distributions across those with and without OLD. Most of the participants were male (72.5%) with a mean age of 56.8 and 54.9% identified as black, non-Hispanic, 43.1% as white or Hispanic/Latino, and 2.0% identified as Asian or Pacific Islander. Most participants were receiving antiretroviral treatment (ART) (92.2%) at the time of study. Smoking status was similar between those with and without OLD, with 52.9% actively smoking at the time of enrollment, however, average pack years were greater in those with OLD (31.1) vs those without OLD (15.2). Lung function ranged from 21 to 128% of predicted normal. Among those with OLD, the average FEV1pp was 67.5% and for those without OLD the average was 104%.

**Table 1:** Demographics of study participant cohort.

	OLD (N = 25)	Without OLD (N = 26)	Total (N = 51)
Sex			
Male	19 (76.0%)	18 (69.2%)	37 (72.5%)
Female	6 (24.0%)	8 (30.8%)	14 (27.5%)
Age			
Mean (SD)	60.0 (8.47)	53.8 (7.30)	56.8 (8.42)
Median [min, max]	58.0 [44.0, 80.0]	54.0 [42.0, 76.0]	56.0 [42.0, 80.0]
Ethnicity			
Black, Non-Hispanic	16 (64.0%)	12 (46.2%)	28 (54.9%)
White, Hispanic/Latino	9 (36.0%)	13 (50.0%)	22 (43.1%)
Asian/pacific islander	0 (0%)	1 (3.8%)	1 (2.0%)
Smoking status			
Former	9 (36.0%)	7 (26.9%)	16 (31.4%)
Never	3 (12.0%)	5 (19.2%)	8 (15.7%)
Yes	13 (52.0%)	14 (53.8%)	27 (52.9%)
Pack years			
Mean (SD)	31.1 (28.9)	15.2 (13.7)	23.0 (23.7)
Median [min, max]	29.2 [0, 120]	13.6 [0, 38.0]	17.0 [0, 120]
Receiving antiretroviral treatment			
Yes	23 (92.0%)	24 (92.3%)	47 (92.2%)
No	2 (8.0%)	2 (7.7%)	4 (7.8%)
FEV1-percent-predicted			
Mean (SD)	67.5 (16.1)	104 (11.2)	85.9 (22.8)
Median [min, max]	68.2 [21.0, 90.4]	102 [81.3, 128]	87.1 [21.0, 128]
FEV1			
Mean (SD)	2.05 (0.600)	3.25 (0.746)	2.66 (0.905)
Median [min, max]	2.00 [0.650, 3.29]	3.08 [1.95, 4.77]	2.59 [0.650, 4.77]
FEV1/FVC			
Mean (SD)	0.553 (0.115)	0.795 (0.0567)	0.676 (0.151)
Median [min, max]	0.588 [0.293, 0.679]	0.789 [0.689, 0.905]	0.689 [0.293, 0.905]
DLCO-percent-predicted			
Mean (SD)	70.5 (26.5)	76.6 (23.1)	73.7 (24.7)
Median [min, max]	57.1 [36.3, 139]	74.5 [14.4, 117]	73.3 [14.4, 139]
M issing	6 (24.0%)	5 (19.2%)	11 (21.6%)
Viral load			
< 50 copies	12 (48.0%)	18 (69.2%)	30 (58.8%)
> 50 copies	1 (4.0%)	3 (11.5%)	4 (7.8%)
Missing	12 (48.0%)	5 (19.2%)	17 (33.3%)

*FEV* forced expiratory volume in 1-s, FVC forced vital capacity, *DLCO* diffusing capacity of lung for carbon monoxide

## Proteases associated with lung function

**Fig. 2:**
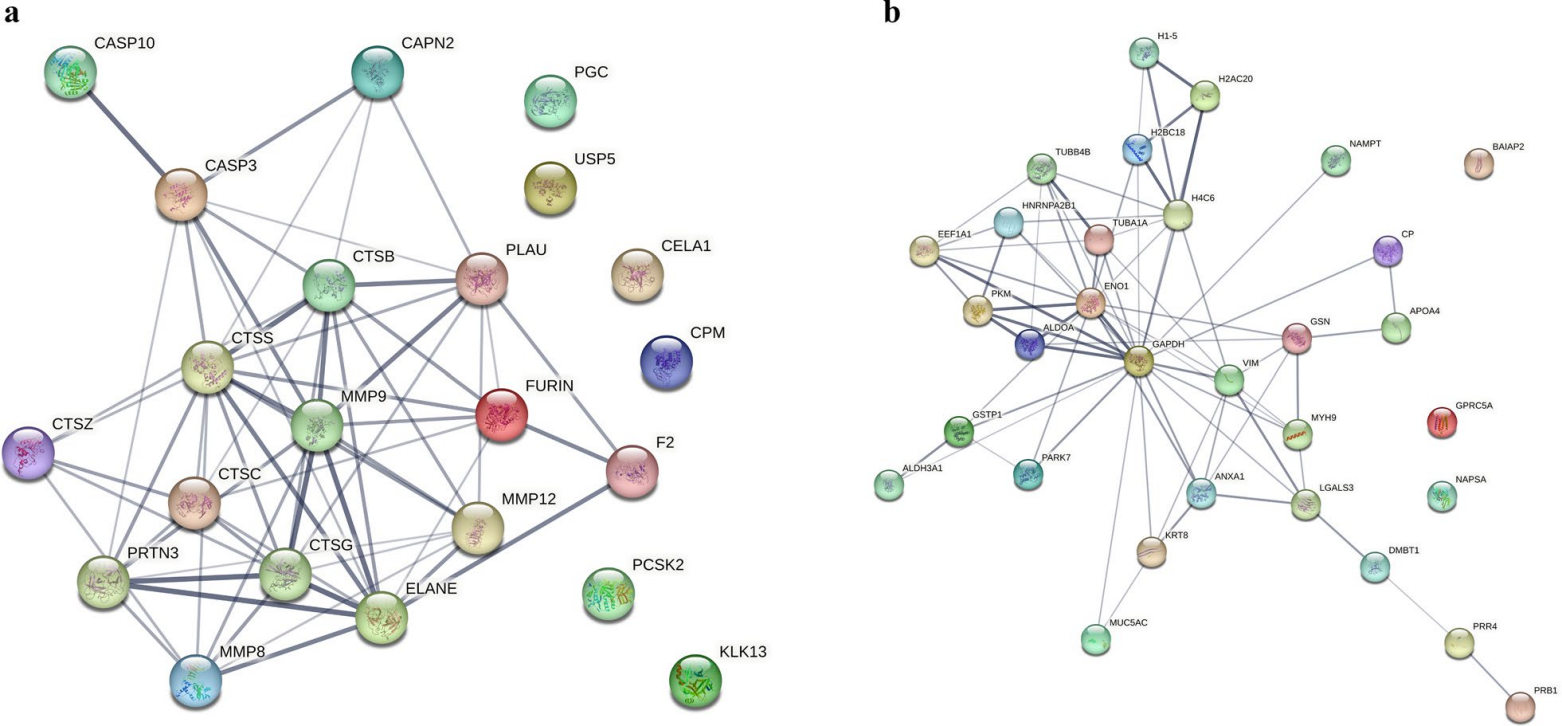
a) STRING diagram demonstrating protein–protein associations of the 26 proteases identified in BALF by LC–MS/MS and SomaScan that associate with FEV1pp. b) STRING diagram demonstrating protein–protein associations of the 31 substrate proteins mapped to endogenous peptides that associate with FEV1pp

To enhance our proteomic coverage to identify proteases in BALF, we leveraged the previously-reported SomaScan proteomic data from the BALF soluble component, along with proteins measured by TMT with MS of both the soluble and insoluble BALF components. [18] A total of 101 proteases were identified, many of which overlapped between the three different methods of measuring proteins (Fig. 1, Table 1S). Of these proteases, 40 were unique to Somascan, 9 unique to the insoluble component of BALF and 3 in the soluble BALF component measured by TMT. Most of these proteases make up a network that is functionally associated or linked (Fig. 2S). We identified 22 proteases that were associated with FEV1pp, four positively correlated and 18 negatively correlated (Table 2). The four proteases that correlated with higher lung function included carboxypeptidase M, prothrombin, urokinase-type plasminogen activator and gastricsin. Many of the 22 proteases associated with lower lung function are proteases previously described in OLD, including cathepsins, metalloproteinases (MMP), caspases and neutrophil elastase. All but six of these proteases have functional associations with each other (Fig. 2a).

**Table 2:** Proteases significantly correlated with FEV1pp across the Somascan and two tandem mass tagging datasets.

UniProt ID	Protease Name	Mean correlation with FEV1pp	Combined p-value	FDR
Proteases with positive associations with FEV1pp				
P14384	Carboxypeptidase M	0.4441	0.0000	0.0000
P00734	Prothrombin	0.3752	0.0000	0.0007
P00749	Urokinase-type plasminogen activator	0.3836	0.0004	0.0054
P20142	Gastricsin	0.3898	0.0007	0.0061
Proteases with negative associations with FEV1pp				
P24158	Myeloblastin	−0.4621	0.0000	0.0002
Q9UKR3	Kallikrein-13	−0.3275	0.0000	0.0002
P07858	Cathepsin B	−0.3481	0.0001	0.0017
P53634	Dipeptidyl peptidase 1	−0.3155	0.0002	0.0028
Q9UNI1	Chymotrypsin-like elastase	−0.4757	0.0004	0.0053
Q9UBR2	Cathepsin Z	−0.2607	0.0006	0.0059
P14780	MMP9	−0.3391	0.0005	0.0059
P22894	MMP8	−0.3268	0.0011	0.0091
P08311	Cathepsin G	−0.3155	0.0014	0.0103
P42574	Caspase-3	−0.3534	0.0014	0.0103
P16519	Neuroendocrine convertase 2	−0.3044	0.0020	0.0136
P17655	Calpain-2 catalytic subunit	−0.1639	0.0036	0.0177
P25774	Cathepsin S	−0.2411	0.0033	0.0177
P08246	Neutrophile Elastase	−0.2988	0.0030	0.0177
P45974	Ubiquitin carboxyl-terminal hydrolase 5	−0.0465	0.0034	0.0177
P39900	Macrophage metalloelastase	−0.4033	0.0030	0.0177
P09958	Furin endoprotease	−0.3957	0.0037	0.0177
Q92851	Caspase-10	–0.0646	0.0061	0.0280

Proteases are ordered by FDR. Note that degree of correlation with FEV1pp and FDR magnitude do not necessarily align

### Protein substrates subject to proteolytic cleavage

**Fig. 3:**
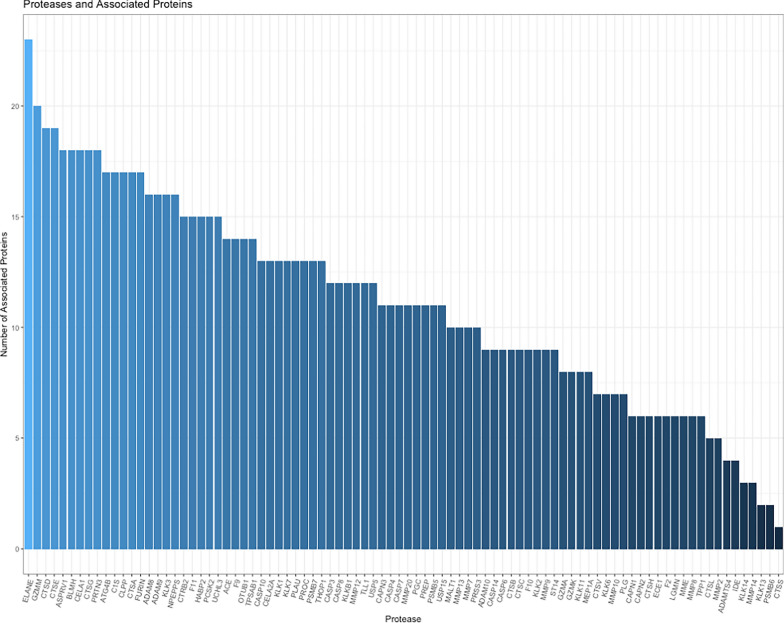
Proteases targeting proteins whose degradation was associated with FEV1pp and the total number of substrate proteins mapped to the endogenous peptides

We identified 31 proteins, mapped from endogenous peptides, that were the substrates for proteolytic cleavage and associated with FEV1pp (Table 3). Table 3 depicts the top 15 proteins and the mean FEV1pp among participants for whom their samples contained these substrate proteins. Among the top five proteins were alpha-enolase, an enzyme involved in glycolysis, histones, and tubulin. Among these 31 proteins, 28 proteins showed inverse relationships with FEV1pp, i.e. increased degradation was associated with lower average FEV1pp, indicating these proteins were more likely to be subject to proteolysis in the presence of OLD. Figure 3a depicts the protein–protein interaction of these 31 proteins and all but three have functional associations. The top ten pathways reflected among these 31 proteins are shown in Table 4. There were 39 pathways with FDR below 0.05, including pathways involving small ubiquitin-like modifier mediated modification (SUMOylation), a post-translational process to control protein quality [33] and histone methylation.

**Table 3:** Top 15 proteins identified from endogenous peptides whose degradation was associated with FEV1pp

Protein	UniProt ID	Mean FEV1pp (Present)	Mean FEV1pp (Absent)	P-Value	FDR
Alpha-enolase	P06733	53.9625	91.8787	0.0004	0.0247
Gelsolin	P06396	45.4126	90.3352	0.0008	0.0247
Histone H4	P62805	61.1725	91.9697	0.0009	0.0247
Tubulin beta-4B chain	P68371	55.6507	90.7484	0.0022	0.0414
Histone H2B type 2-F	Q5QNW6	56.8110	91.3487	0.0036	0.0414
Tubulin alpha-1A chain	Q71U36	64.6865	91.1127	0.0045	0.0414
Glyceraldehyde-3-phosphate dehydrogenase	P04406	65.3887	91.5802	0.0046	0.0414
Myosin-9	P35579	61.5678	88.5792	0.0046	0.0414
Aldehyde dehydrogenase, dimeric NADP-preferring	P30838	61.6220	91.1401	0.0046	0.0414
Glutathione S-transferase P	P09211	61.7737	91.1076	0.0054	0.0414
Vimentin	P08670	78.8246	96.0831	0.0054	0.0414
Isoform A2 of Heterogeneous nuclear ribonucleoproteins A2/B1	P22626-2	66.1016	89.0858	0.0062	0.0425
Galectin-3	P17931	70.2732	92.4552	0.0066	0.0425
Parkinson disease protein 7	Q99497	62.8499	90.8770	0.0074	0.0442
Basic salivary proline-rich protein 1	P04280	66.1060	90.7664	0.0087	0.0486

**Table 4:** Pathways reflected in proteins whose degradation was associated with FEV1pp

Pathway	Number overlapping genes	Number pathway genes	P-value	Q-value
SUMO E3 ligases SUMOylate target proteins	15	15 (177)	4.94E-06	0.00745
RMTs methylate histone arginines	15	15 (74)	4.94E-06	0.00745
SUMOylation	15	15 (182)	4.94E-06	0.00745
SUMOylation of chromatin organization proteins	14	14 (72)	1.27E-05	0.00745
HDMs demethylate histones	14	14 (50)	1.27E-05	0.00745
PKMTs methylate histone lysines	14	14 (73)	1.27E-05	0.00745
Deposition of new CENPA-containing nucleosomes at the centromere	14	14 (43)	1.27E-05	0.00745
Nucleosome assembly	14	14 (43)	1.27E-05	0.00745
HDACs deacetylate histones	16	17 (94)	2.08E-05	0.00883
HCMV Early Events	16	17 (109)	2.08E-05	0.00883

### Proteases participating in substrate cleavage

To identify the proteases linked to the generation of the endogenous peptides, we analyzed 101 proteases identified across the SomaScan and TMT datasets with the top 31 identified substrate proteins that associated with FEV1pp. After linking candidate endogenous peptides to their corresponding proteases responsible for their cleavage by examining the z-scores, we studied how many proteins each protease cleaved. The number of proteins assigned to each protease ranged from one to 23 (Fig. 3; Table 2S) with the top 10 proteases included neutrophil elastase, granzyme, and cathepsin D (Table 5).

**Table 5:** Top 10 most active proteases mapped to cleaved proteins. P-value and FDR describes significance of correlation between protease and FEV1pp.

Gene	Protease	No. Proteins	p-Value	FDR
ELANE	Neutrophil elastase	23	0.003	0.018
GZMM	Granzyme M	20	0.245	0.420
CTSD	Cathepsin D	19	0.233	0.412
CTSE	Cathepsin E	19	0.154	0.330
ASPRV1	Aspartic peptidase	18	0.166	0.346
BLMH	Bleomycin hydrolase	18	0.405	0.594
CELA1	Chymotrypsin like elastase 1	18	0.000	0.005
CTSG	Cathepsin G	18	0.001	0.010
PRTN3	Proteinase 3	18	0.000	0.000
ATG4B	Autophagy related 4B cysteine peptidase	17	0.785	0.890

Proteases are ordered by number of associated proteins cleaved

## Discussion

Proteases are a diverse group of proteins comprising over 500 members which makes up almost 2% of the human genome. There are five major classes of proteases in mammals with serine, cysteine and metallo- proteases being the most prevalent in human lung disease. Traditionally these proteases have been viewed as substrate specific protein degrading enzymes and originally not to be participants in signaling or regulatory pathways. In the last decade, advances in degradomics and the study of protease substrate have revealed that protease targets and their substrates are complex [34, 35]. It is now evident that proteases are key components of regulatory mechanisms via cleavage of specific substrates with concomitant activation, silencing or modulation of regulatory functions through a mechanism called proteolytic processing [34]. While most studies related to the role of proteases in OLD, both HIV and non-HIV associated, have been limited to individual proteases or protease families; it is highly unlikely that single proteases or even single protease families are solely responsible for OLD pathogenesis. More likely there are complex interactions among proteases and their substrates that participate in multiplexed regulatory systems. In this study, we characterized the complex protease proteome in HIV-associated OLD via a combination of proteome profiling and identified protease activity and their substrates through peptidomic analysis.

Utilizing a comprehensive proteomic approach that included a combination of targeted aptamer-based proteomics and untargeted mass spectrometry with TMT labeling we identified 101 proteases within the BALF in PLWH, 22 of which were significantly associated with lung function as measured by FEV1pp. Proteases are key regulatory proteins in both homeostasis and disease and several of the proteases we identified are associated with normal lung function. One protease, gastricsin, is a gastric protease and likely represents micro-aspiration, which is common in individuals with OLD [36]. Interestingly, gastricsin was observed in individuals with preserved FEV1. Aspiration is likely to be equally, if not more, common in those with severe lung function, although it is probably less prevalent compared to the proteases that are upregulated in disease. We found both prothrombin and urokinase-type plasminogen activator to be associated with normal lung function and these proteases have roles in fibrin homeostasis in the healthy lung [37, 38]. Many of the proteases associated with lower lung function have been described in OLD, such as the metalloproteinases, cathepsins, caspases and neutrophil elastase. Caspases are proteases involved in apoptosis and associated with the generation of emphysema [39]. Unfortunately, we were not able to correlate specific proteases with emphysema in this cohort as CT imaging was limited.

What is most striking is that no single protease or protease family predominates. Rather, there is upregulation of many proteases across divergent protease families. Proteases can interact either directly or indirectly with other proteases and become interconnected in what has been termed a ‘protease web’ [40]. We found that all but six of the proteases that associated with lung function were part of such a functionally-associated network. This interconnection and redundancies of proteases in OLD create challenges in identifying individual therapeutic targets for anti-protease therapy. Most of the proteases belong to a common network and it remains unknown whether it requires targeting individual or multiple proteases to effectively block proteolytic activity. Proteases are also involved in normal physiological functions; therefore, broad proteolytic blockage could have untoward effects on homeostasis.

Proteases initiate and modulate many important cellular functions by highly specific substrate cleavage. In the inflammatory state, upregulated proteases have a wide range of substrate targets that are not limited to extracellular matrix proteins. Not only do proteases cleave multiple substrates, but substrates can be cleaved by multiple proteases. Utilizing peptidomic analysis by mass spectrometry, we were able to map endogenously produced peptides to their cognate proteins. All but three of these proteins had functional associations, suggesting the targeting or susceptibility of specific biological pathways. In addition, most of these proteins were associated with lower lung function and mapped to pathways vital for cellular function, including SUMOylation. SUMOylation is critical in broad biological functions including cell cycle and protein stability. Cigarette smoke upregulates SUMOylation in human bronchial epithelial cells, providing a potential link to OLD [41].

Although our complementary proteomics analysis identified proteases that had upregulated abundance with disease, increased abundance does not guarantee increased protease activity. To address this, we utilized endogenous peptides to characterize protease activity. To identify the proteases responsible for protein degradation we assigned cleavage sites to endogenously produced peptides and matched these sites to their conjugate proteases. Neutrophil elastase, granzyme M and cathepsins D and E were among the most active proteases linked to substrate degradation. These proteases are commonly associated with OLD [42]. Although metalloproteinase and caspase proteases were upregulated in disease, they were less active.

Limitations of this study include the relatively small sample size, lack of non-HIV controls and lack of more detailed lung structure and function metrics, (e.g. CT quantitative imaging). In addition, there were a large percentage of smokers, current or past, in both those with normal lung function and disease. Cigarette smoking is associated with elevated protease activity in both humans and animal models and additional studies are needed to differentiate the roles of tobacco smoke and HIV infection in protease activation [43]. Overall, this study brings to light the large repertoire of proteases that are upregulated and actively involved in proteolysis in HIV-associated OLD. In addition, we identified specific proteins that were subject to proteolysis that are linked to specific pathways vital to cellular and organ function, suggesting a possible role in pathogenesis Future studies are needed to validate these findings, especially comparing HIV to non-HIV controls. It is important for future mechanistic studies to be aware of the multitude of proteases and their substrates that are active in HIV-associated OLD.

## Supplementary Information

Supplementary material 1.

Supplementary material 2.

## Data Availability

No datasets were generated or analysed during the current study.
