# Supplementary material for: Novel approach to exploring protease activity and targets in HIV-associated obstructive lung disease using combined proteomic-peptidomic analysis
Source: Respir Res. 2024 Sep 10;25:337. doi: 10.1186/s12931-024-02933-9 (PMC11385845; doi:10.1186/s12931-024-02933-9)
Supplement: Supplementary file 1 — Supplementary material 1. [file 12931_2024_2933_MOESM1_ESM.docx]

**Supplement**

**Methods Supplement:**

**Data Cleaning:** Prior to statistical analysis, we removed non-human proteins from the dataset. We applied a log(1+x) transformation to the MaxLFQ protein intensity values to normalize their distributions. Sample processing yielded four datasets which we included in our analysis: a targeted SomaScan aptamer-based proteomics dataset obtained from the BALF soluble component, an untargeted tandem mass tagging proteomics dataset obtained from the BALF soluble component, an untargeted tandem mass tagging proteomics dataset obtained from the BALF insoluble component, and a mass spectrometry dataset describing peptide abundances in the BALF soluble component. Endogenous peptides were assigned to their corresponding proteins from which they were cleaved. Our analysis of the peptides occurred at the protein level, i.e. we focused on the abundances of the degraded proteins, instead of the individual peptides. We now describe the data cleaning process for each of the four datasets.

Our data cleaning approach for the SomaScan dataset has been described previously.[1] Briefly, we removed aptamers whose abundances fell below an empirical limit of detection, applied a log(1+x) transformation to the abundances, and centered and scaled each aptamer’s abundance to mean 0 and standard deviation 1 to normalize their variances and facilitate comparisons.

We applied the same cleaning procedure to the soluble component tandem mass tagging and the insoluble component tandem mass tagging datasets. During mass spectrometry, samples were run on three plexes. For each dataset, we removed proteins not detected on at least two plexes. We applied a $log(1+x)$ transformation to the protein abundances. We then imputed missing abundances among proteins not detected on one plex using a singular value decomposition-based imputation approach with four components.[2] We applied ComBat to each dataset separately to adjust for plex-based differences.[3] We then centered and scaled each protein to have mean 0 and standard deviation 1.

To clean the peptides data, we excluded any non-human proteins. We applied a log(1+x) transformation to the MaxLFQ protein intensity values. We did not implement any additional standardization of the protein intensities.

| **Protease UniProt** | **Mean Correlation** | **Combined P-Value** | **FDR** |
| --- | --- | --- | --- |
| P14384 | 0.4441 | 0.0000 | 0.0000 |
| P24158 | -0.4621 | 0.0000 | 0.0002 |
| Q9UKR3 | -0.3275 | 0.0000 | 0.0002 |
| P00734 | 0.3752 | 0.0000 | 0.0007 |
| P07858 | -0.3481 | 0.0001 | 0.0017 |
| P53634 | -0.3155 | 0.0002 | 0.0028 |
| Q9UNI1 | -0.4757 | 0.0004 | 0.0053 |
| P00749 | 0.3836 | 0.0004 | 0.0054 |
| Q9UBR2 | -0.2607 | 0.0006 | 0.0059 |
| P14780 | -0.3391 | 0.0005 | 0.0059 |
| P20142 | 0.3898 | 0.0007 | 0.0061 |
| P22894 | -0.3268 | 0.0011 | 0.0091 |
| P08311 | -0.3155 | 0.0014 | 0.0103 |
| P42574 | -0.3534 | 0.0014 | 0.0103 |
| P16519 | -0.3044 | 0.0020 | 0.0136 |
| P17655 | -0.1639 | 0.0036 | 0.0177 |
| P25774 | -0.2411 | 0.0033 | 0.0177 |
| P08246 | -0.2988 | 0.0030 | 0.0177 |
| P45974 | -0.0465 | 0.0034 | 0.0177 |
| P39900 | -0.4033 | 0.0030 | 0.0177 |
| P09958 | -0.3957 | 0.0037 | 0.0177 |
| Q92851 | -0.0646 | 0.0061 | 0.0280 |
| P20231 | -0.3442 | 0.0125 | 0.0547 |
| Q99538 | -0.1451 | 0.0172 | 0.0695 |
| O14773 | -0.2167 | 0.0166 | 0.0695 |
| P15144 | -0.0621 | 0.0259 | 0.0967 |
| O14672 | 0.0214 | 0.0253 | 0.0967 |
| P10619 | -0.2606 | 0.0299 | 0.1079 |
| P27487 | 0.2467 | 0.0472 | 0.1645 |
| O60911 | -0.0555 | 0.0512 | 0.1667 |
| Q9UI42 | -0.1268 | 0.0510 | 0.1667 |
| P09960 | 0.0246 | 0.0616 | 0.1943 |
| P09238 | -0.2198 | 0.0658 | 0.1958 |
| P45452 | -0.2570 | 0.0659 | 0.1958 |
| P49862 | -0.1649 | 0.0783 | 0.2208 |
| P55210 | -0.1072 | 0.0813 | 0.2208 |
| Q14790 | 0.2612 | 0.0831 | 0.2208 |
| Q9UDY8 | 0.2433 | 0.0822 | 0.2208 |
| P08253 | -0.2408 | 0.0855 | 0.2213 |
| P14735 | -0.0251 | 0.1083 | 0.2736 |
| P03952 | 0.1729 | 0.1237 | 0.2906 |
| Q9UBX7 | -0.1698 | 0.1220 | 0.2906 |
| Q92876 | -0.1943 | 0.1182 | 0.2906 |
| P20807 | 0.2106 | 0.1340 | 0.3077 |
| P06870 | -0.2226 | 0.1417 | 0.3110 |
| P35030 | -0.1333 | 0.1400 | 0.3110 |
| P14091 | 0.2007 | 0.1537 | 0.3303 |
| Q53RT3 | 0.1949 | 0.1661 | 0.3464 |
| P12544 | 0.0074 | 0.1681 | 0.3464 |
| P15169 | -0.1374 | 0.1723 | 0.3481 |
| Q9NQH7 | 0.1875 | 0.1833 | 0.3629 |
| P09668 | -0.0153 | 0.1967 | 0.3821 |
| P08217 | 0.1803 | 0.2008 | 0.3827 |
| Q96IY4 | -0.0242 | 0.2150 | 0.4021 |
| P78325 | 0.1724 | 0.2217 | 0.4071 |
| P28074 | -0.0582 | 0.2303 | 0.4122 |
| P07339 | -0.1684 | 0.2326 | 0.4122 |
| P12821 | 0.1256 | 0.2422 | 0.4197 |
| P51124 | 0.1641 | 0.2451 | 0.4197 |
| O43897 | -0.1626 | 0.2495 | 0.4199 |
| P16870 | -0.1128 | 0.2660 | 0.4404 |
| P15374 | 0.1553 | 0.3083 | 0.5013 |
| P08473 | -0.1428 | 0.3127 | 0.5013 |
| Q9UHL4 | 0.0159 | 0.3523 | 0.5560 |
| Q96FW1 | 0.1400 | 0.3592 | 0.5580 |
| P20151 | 0.1283 | 0.3646 | 0.5580 |
| Q9NQW7 | -0.1338 | 0.3810 | 0.5744 |
| Q13867 | -0.0042 | 0.4047 | 0.5940 |
| P50281 | -0.1177 | 0.4058 | 0.5940 |
| Q6UB28 | -0.1086 | 0.4435 | 0.6309 |
| P00740 | 0.1208 | 0.4428 | 0.6309 |
| P00747 | 0.1232 | 0.4596 | 0.6359 |
| P03951 | -0.1280 | 0.4573 | 0.6359 |
| Q9Y4E8 | 0.1148 | 0.4754 | 0.6489 |
| P07288 | 0.0976 | 0.4913 | 0.6616 |
| Q16819 | -0.0951 | 0.5535 | 0.7356 |
| Q13443 | 0.0799 | 0.5733 | 0.7520 |
| Q14520 | 0.1089 | 0.5829 | 0.7548 |
| Q9Y5Y6 | -0.0754 | 0.6227 | 0.7765 |
| P52888 | -0.0776 | 0.6126 | 0.7765 |
| P42892 | -0.0827 | 0.6214 | 0.7765 |
| P09871 | -0.0672 | 0.6637 | 0.8175 |
| P31944 | 0.0785 | 0.7017 | 0.8337 |
| P07384 | 0.0532 | 0.6962 | 0.8337 |
| P55786 | -0.0613 | 0.6892 | 0.8337 |
| Q16740 | -0.0442 | 0.7557 | 0.8875 |
| P49863 | 0.0425 | 0.7648 | 0.8879 |
| P07711 | -0.0385 | 0.8019 | 0.8900 |
| P48147 | -0.0387 | 0.8009 | 0.8900 |
| Q9Y4P1 | 0.0387 | 0.7852 | 0.8900 |
| O60882 | 0.0364 | 0.7976 | 0.8900 |
| P09237 | -0.0341 | 0.8475 | 0.9189 |
| P55212 | 0.0307 | 0.8411 | 0.9189 |
| P04070 | 0.0259 | 0.8552 | 0.9189 |
| Q9P0G3 | 0.0430 | 0.8955 | 0.9520 |
| Q6GPI1 | -0.0165 | 0.9076 | 0.9548 |
| P49662 | 0.0134 | 0.9251 | 0.9633 |
| P28072 | 0.0240 | 0.9487 | 0.9778 |
| P00742 | -0.0027 | 0.9896 | 0.9961 |
| Q99436 | 0.0033 | 0.9831 | 0.9961 |
| O75173 | 0.0007 | 0.9961 | 0.9961 |

Table 1S. Proteases identified in the combined proteome measured by SomaScan, TMT labelled MS of soluble and insoluble BALF fractions and their correlation with FEV1pp.

Figure 1S

Figure 1S. Cleavage site assignment. Peptides were assigned cleavage sites categorized based on cleave location as depicted above. a) mapping of cleavage for protease and endopeptidases; b) mapping of cleavage for exopeptidases and c) identification of peptides resulting from secondary cleavage.

Figure 2S.


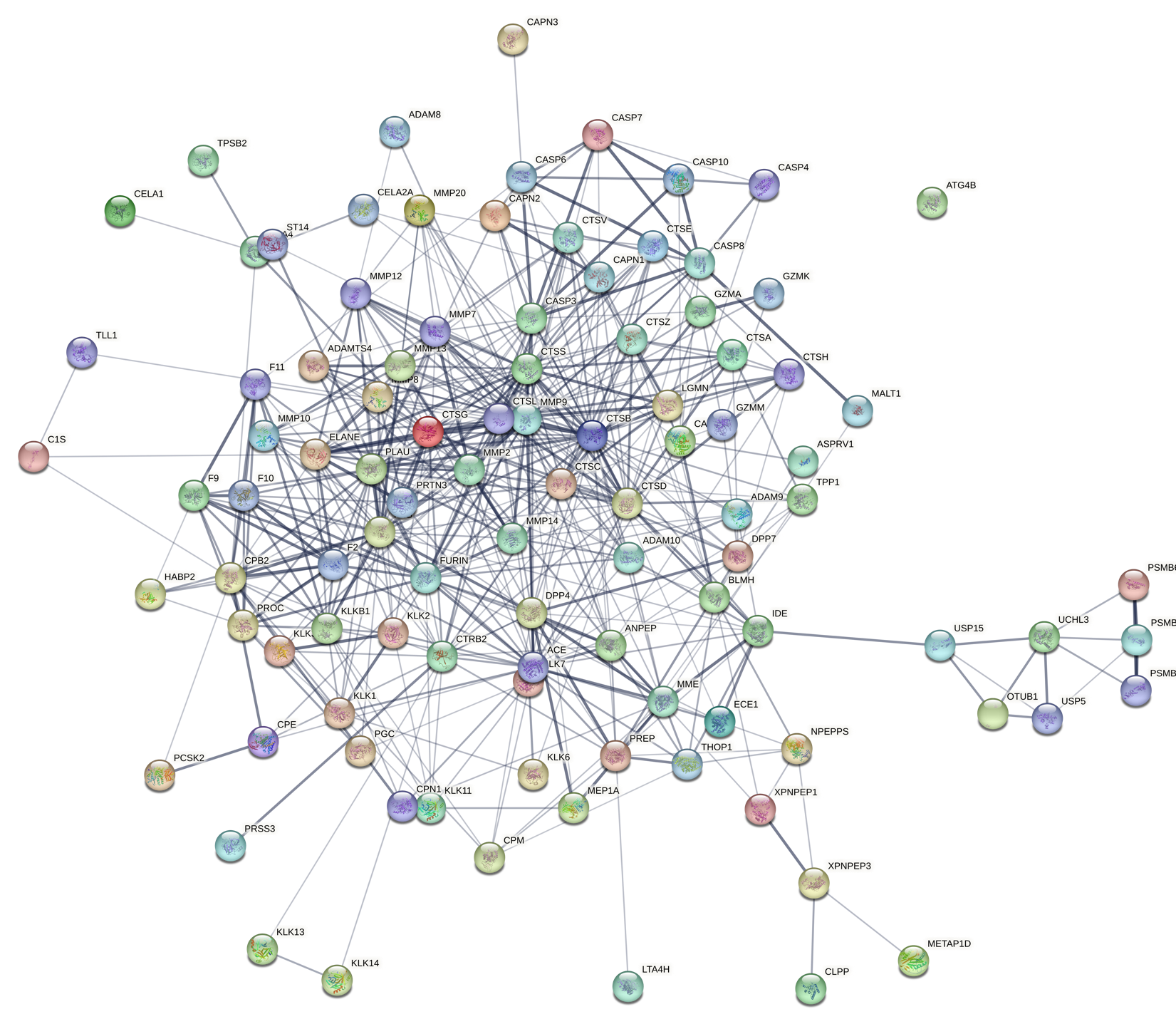


Figure 2S. STRING functional network of proteases identified and quantified in BALF, combining SomaScan with the TMT-labelled MS analysis of the insoluble and soluble BALF fractions.

Table 2S: Proteases and their assigned proteolytic proteins. Protease listed by gene and proteins listed by UniProt number.

References:

1. Samorodnitsky S, Lock EF, Kruk M, Morris A, Leung JM, Kunisaki KM, Griffin TJ, Wendt CH. Lung proteome and metabolome endotype in HIV-associated obstructive lung disease. *ERJ Open Res* 2023: 9(2).

2. Fuentes M, Guttorp P, Sampson P. Using transforms to analyze space-time processes. *In:* Finkenstadt B, Held L, V I, eds. Statistical Methods for Spatio-Temporal Systems. Chapman and Hall CRC, 2006.

3. Johnson WE, Li C, Rabinovic A. Adjusting batch effects in microarray expression data using empirical Bayes methods. *Biostatistics* 2007: 8(1): 118-127.
