# Supplementary material for: Novel approach to exploring protease activity and targets in HIV-associated obstructive lung disease using combined proteomic-peptidomic analysis
Source: Respir Res. 2024 Sep 10;25:337. doi: 10.1186/s12931-024-02933-9 (PMC11385845; doi:10.1186/s12931-024-02933-9)
Supplement: Supplementary file 2 — Supplementary material 2. [file 12931_2024_2933_MOESM2_ESM.docx]

| **Protease** | **No.Proteins** | **Protein 1** | **Protein 2** | **Protein 3** | **Protein 4** | **Protein 5** | **Protein 6** | **Protein 7** |
| --- | --- | --- | --- | --- | --- | --- | --- | --- |
| ELANE | 23 | P30838 | P04075 | P04083 | P00450 | Q9UGM3-2 | P68104 | P06733 |
| GZMM | 20 | P30838 | P04075 | P04083 | P06727 | Q9UGM3-2 | P68104 | P06733 |
| CTSD | 19 | P30838 | P04075 | P04083 | P06727 | P00450 | P06733 | P04406 |
| CTSE | 19 | P30838 | P04075 | P04083 | P06727 | P00450 | P06733 | P04406 |
| ASPRV1 | 18 | P30838 | P04075 | P04083 | P00450 | P06733 | P04406 | P06396 |
| BLMH | 18 | P30838 | P04075 | P04083 | P06727 | P00450 | P68104 | P06733 |
| CELA1 | 18 | P30838 | P04083 | P00450 | P68104 | P06733 | P04406 | Q8NFJ5 |
| CTSG | 18 | P30838 | P04083 | Q9UQB8-5 | P00450 | P68104 | P06733 | P04406 |
| PRTN3 | 18 | P30838 | P06727 | P00450 | Q9UGM3-2 | P68104 | P06733 | P04406 |
| ATG4B | 17 | P04075 | P06727 | P00450 | P68104 | P06733 | P04406 | Q8NFJ5 |
| C1S | 17 | P30838 | P04075 | P04083 | P00450 | P68104 | P04406 | P09211 |
| CLPP | 17 | P30838 | P04083 | P00450 | Q9UGM3-2 | P06733 | P04406 | P06396 |
| CTSA | 17 | P00450 | Q9UGM3-2 | P68104 | P06733 | P04406 | P06396 | P62805 |
| FURIN | 17 | P04075 | P04083 | Q9UQB8-5 | P00450 | Q9UGM3-2 | P68104 | P06733 |
| ADAM8 | 16 | P30838 | P04075 | P04083 | P06727 | P68104 | P04406 | P06396 |
| ADAM9 | 16 | P30838 | P06727 | P68104 | P06733 | P04406 | Q8NFJ5 | P06396 |
| KLK3 | 16 | P30838 | P04075 | P00450 | P06733 | P04406 | Q8NFJ5 | P06396 |
| NPEPPS | 16 | P30838 | P04083 | P06727 | P68104 | P04406 | Q8NFJ5 | P06396 |
| CTRB2 | 15 | P30838 | P04083 | P06727 | P06733 | P04406 | P06396 | P09211 |
| F11 | 15 | P04075 | P00450 | P68104 | P06733 | Q8NFJ5 | P06396 | P09211 |
| HABP2 | 15 | P04075 | P00450 | P68104 | P06733 | P04406 | Q8NFJ5 | P06396 |
| PCSK2 | 15 | P04075 | P04083 | Q9UQB8-5 | P00450 | Q9UGM3-2 | P68104 | P06733 |
| UCHL3 | 15 | P04083 | P06727 | P00450 | P68104 | P06733 | P04406 | Q16777 |
| ACE | 14 | P30838 | P04075 | P06727 | P00450 | P06733 | P04406 | P06396 |
| F9 | 14 | P30838 | P00450 | Q9UGM3-2 | P68104 | P06733 | P04406 | Q8NFJ5 |
| OTUB1 | 14 | P30838 | P04075 | P04083 | P00450 | P68104 | P06733 | P04406 |
| TPSAB1 | 14 | P30838 | P04083 | P68104 | P06733 | P04406 | P06396 | P09211 |
| CASP10 | 13 | P04083 | P00450 | P68104 | P06733 | P04406 | P06396 | P17931 |
| CELA2A | 13 | P30838 | P04075 | P06733 | P04406 | P06396 | Q16777 | P05787 |
| KLK1 | 13 | P04075 | P06727 | P06733 | P09211 | Q16777 | P62805 | P22626-2 |
| KLK7 | 13 | P04083 | P06727 | P00450 | P06733 | P04406 | P09211 | Q16777 |
| PLAU | 13 | P04075 | P00450 | P68104 | P06733 | P04406 | Q8NFJ5 | P06396 |
| PROC | 13 | P04083 | P06727 | P00450 | P06733 | P04406 | P06396 | P22626-2 |
| PSMB7 | 13 | P04075 | P04083 | P06727 | P00450 | Q9UGM3-2 | P68104 | P06733 |
| THOP1 | 13 | P06727 | Q9UQB8-5 | P68104 | P06396 | P09211 | P22626-2 | P05787 |
| CASP3 | 12 | P30838 | P04075 | P00450 | P04406 | P06396 | P17931 | P43490 |
| CASP8 | 12 | P04075 | P00450 | P04406 | P06396 | P35579 | P43490 | Q99497 |
| KLKB1 | 12 | P04083 | P00450 | P06733 | Q8NFJ5 | P06396 | P09211 | Q16777 |
| MMP12 | 12 | P30838 | P04075 | P68104 | P06733 | P04406 | P06396 | P16401 |
| TLL1 | 12 | P04075 | P06733 | P04406 | P06396 | P22626-2 | P98088 | P35579 |
| USP5 | 12 | P30838 | P04075 | P04083 | P06727 | P00450 | P06733 | P04406 |
| CAPN3 | 11 | P04083 | P00450 | Q9UGM3-2 | Q8NFJ5 | P06396 | P98088 | P35579 |
| CASP4 | 11 | P04075 | P04083 | P00450 | P68104 | P04406 | P06396 | P09211 |
| CASP7 | 11 | P30838 | P04075 | P00450 | P04406 | P06396 | P35579 | P43490 |
| MMP20 | 11 | P00450 | P04406 | Q8NFJ5 | P06396 | P09211 | P35579 | O96009 |
| PGC | 11 | P04075 | P06727 | P00450 | P68104 | P04406 | P62805 | P22626-2 |
| PREP | 11 | P30838 | P04075 | Q9UGM3-2 | Q8NFJ5 | Q16777 | O96009 | P14618 |
| PSMB5 | 11 | P04075 | P68104 | P04406 | P16401 | Q16777 | P05787 | P43490 |
| USP15 | 11 | P04075 | P04083 | P06727 | P04406 | P98088 | P35579 | O96009 |
| MALT1 | 10 | P04075 | P04083 | P06733 | P04406 | P06396 | P09211 | Q16777 |
| MMP13 | 10 | P04075 | P68104 | Q8NFJ5 | P06396 | P22626-2 | P98088 | P35579 |
| MMP7 | 10 | P30838 | P04075 | P00450 | P68104 | P04406 | P06396 | Q16777 |
| PRSS3 | 10 | P04083 | P00450 | Q9UGM3-2 | P06733 | P04406 | P35579 | P14618 |

| ADAM10 | 9 | P04083 | P00450 | P04406 | P06396 | P62805 | P17931 | P35579 |
| --- | --- | --- | --- | --- | --- | --- | --- | --- |
| CASP14 | 9 | P04075 | P04083 | P00450 | P68104 | P04406 | P06396 | P98088 |
| CASP6 | 9 | P30838 | P04083 | P68104 | P04406 | P09211 | P22626-2 | P43490 |
| CTSB | 9 | P30838 | P04075 | P00450 | P04406 | P16401 | P22626-2 | P98088 |
| CTSC | 9 | P00450 | P04406 | Q8NFJ5 | P62805 | P14618 | Q16378 | Q71U36 |
| F10 | 9 | P04075 | P00450 | P68104 | P04406 | P06396 | P09211 | Q16777 |
| KLK2 | 9 | P30838 | P00450 | P68104 | P16401 | Q16777 | P62805 | P22626-2 |
| MMP9 | 9 | P04075 | P68104 | P06733 | Q8NFJ5 | P22626-2 | P17931 | P35579 |
| ST14 | 9 | P04083 | P68104 | P04406 | P06396 | P62805 | P35579 | Q99497 |
| GZMA | 8 | P04075 | P06733 | P06396 | P09211 | Q16777 | Q5QNW6 | P17931 |
| GZMK | 8 | P30838 | P04075 | P00450 | P06733 | P09211 | P17931 | Q71U36 |
| KLK11 | 8 | P30838 | P04075 | P06733 | P04406 | Q8NFJ5 | P16401 | P35579 |
| MEP1A | 8 | P04075 | P04083 | P06733 | P04406 | P35579 | Q16378 | P68371 |
| CTSV | 7 | P68104 | P06733 | P35579 | O96009 | P14618 | P68371 | P08670 |
| KLK6 | 7 | P30838 | P00450 | P68104 | P16401 | Q16777 | P22626-2 | P05787 |
| MMP10 | 7 | P04075 | P00450 | P06733 | P06396 | P35579 | O96009 | Q99497 |
| PLG | 7 | P04075 | Q8NFJ5 | P06396 | P09211 | P05787 | P14618 | Q16378 |
| CAPN1 | 6 | Q8NFJ5 | P09211 | P35579 | P14618 | P04280 | P68371 |  |
| CAPN2 | 6 | Q8NFJ5 | P09211 | P35579 | P14618 | P04280 | Q71U36 |  |
| CTSH | 6 | P04406 | P35579 | Q99497 | P14618 | Q16378 | P68371 |  |
| ECE1 | 6 | P30838 | P04406 | P06396 | Q16777 | P17931 | P35579 |  |
| F2 | 6 | Q9UGM3-2 | P06733 | P06396 | P22626-2 | P17931 | Q71U36 |  |
| LGMN | 6 | P04083 | Q16777 | P62805 | P35579 | P43490 | O96009 |  |
| MME | 6 | P06727 | P00450 | P06733 | P04406 | P35579 | P08670 |  |
| MMP8 | 6 | P04075 | P68104 | P06733 | P06396 | P35579 | P04280 |  |
| TPP1 | 6 | P30838 | P04075 | P00450 | P68104 | Q16378 | P68371 |  |
| CTSL | 5 | Q8NFJ5 | P06396 | P05787 | P35579 | P14618 |  |  |
| MMP2 | 5 | P04075 | P06396 | P35579 | O96009 | Q71U36 |  |  |
| ADAMTS4 | 4 | P68104 | P06733 | P06396 | P62805 |  |  |  |
| IDE | 4 | P06727 | Q16777 | P14618 | Q71U36 |  |  |  |
| KLK14 | 3 | P06733 | Q8NFJ5 | P22626-2 |  |  |  |  |
| MMP14 | 3 | P06733 | P06396 | P14618 |  |  |  |  |
| KLK13 | 2 | P00450 | P09211 |  |  |  |  |  |
| PSMB6 | 2 | P06733 | P08670 |  |  |  |  |  |
| CTSS | 1 | P06396 |  |  |  |  |  |  |

| **Protein 8** | **Protein 9** | **Protein 10** | **Protein 11** | **Protein 12** | **Protein 13** | **Protein 14** | **Protein 15** | **Protein 16** |
| --- | --- | --- | --- | --- | --- | --- | --- | --- |
| P04406 | P06396 | P09211 | Q16777 | P62805 | P22626-2 | P17931 | P98088 | P35579 |
| P04406 | P06396 | P16401 | Q16777 | P62805 | P05787 | P35579 | O96009 | Q99497 |
| P09211 | P16401 | Q16777 | P62805 | P05787 | P35579 | P43490 | O96009 | P14618 |
| P06396 | P09211 | P16401 | Q16777 | P22626-2 | P05787 | P98088 | P35579 | P43490 |
| P09211 | P62805 | P22626-2 | P05787 | P17931 | P35579 | P43490 | O96009 | Q71U36 |
| P04406 | P06396 | P22626-2 | P05787 | P35579 | P43490 | O96009 | P14618 | Q71U36 |
| P06396 | P09211 | P62805 | P22626-2 | P05787 | P98088 | P35579 | P43490 | P14618 |
| Q8NFJ5 | P06396 | P16401 | P22626-2 | P35579 | P43490 | O96009 | P14618 | Q71U36 |
| P06396 | P09211 | Q16777 | P22626-2 | P05787 | P17931 | P35579 | P43490 | Q99497 |
| P06396 | P09211 | P16401 | P22626-2 | P05787 | P35579 | P43490 | O96009 | Q71U36 |
| Q16777 | P17931 | P35579 | P43490 | Q99497 | P14618 | Q16378 | Q71U36 | P68371 |
| P09211 | Q16777 | P17931 | P98088 | P35579 | P43490 | O96009 | P14618 | Q71U36 |
| P17931 | P98088 | P35579 | P43490 | O96009 | P14618 | Q16378 | Q71U36 | P68371 |
| P04406 | P06396 | Q16777 | P62805 | P05787 | P35579 | P14618 | Q71U36 | P68371 |
| Q16777 | P22626-2 | P05787 | P35579 | Q99497 | P14618 | Q71U36 | P68371 | P08670 |
| P09211 | Q16777 | P22626-2 | P05787 | P35579 | O96009 | P14618 | P68371 | P08670 |
| P09211 | P22626-2 | P05787 | O96009 | Q99497 | P04280 | Q71U36 | P68371 | P08670 |
| Q16777 | P62805 | P17931 | P35579 | P43490 | P14618 | Q71U36 | P68371 | P08670 |
| P22626-2 | P17931 | P35579 | O96009 | P14618 | Q71U36 | P68371 | P08670 |  |
| P16401 | Q16777 | P17931 | P35579 | P43490 | O96009 | P14618 | P68371 |  |
| P09211 | Q16777 | P62805 | P22626-2 | P35579 | P43490 | P14618 | Q71U36 |  |
| P04406 | P06396 | P22626-2 | P05787 | P17931 | P35579 | P68371 | P08670 |  |
| P62805 | P98088 | P35579 | O96009 | P14618 | Q71U36 | P68371 | P08670 |  |
| P62805 | P22626-2 | P35579 | O96009 | P14618 | Q71U36 | P08670 |  |  |
| P06396 | P16401 | Q16777 | P22626-2 | P35579 | P04280 | P68371 |  |  |
| P06396 | P35579 | P43490 | P14618 | Q71U36 | P68371 | P08670 |  |  |
| P16401 | P62805 | P05787 | P14618 | Q71U36 | P68371 | P08670 |  |  |
| P35579 | P43490 | P14618 | Q71U36 | P68371 | P08670 |  |  |  |
| P17931 | P35579 | P43490 | P14618 | Q71U36 | P68371 |  |  |  |
| P05787 | P35579 | Q99497 | P14618 | Q71U36 | P08670 |  |  |  |
| P22626-2 | P43490 | P14618 | Q71U36 | P68371 | P08670 |  |  |  |
| P22626-2 | P17931 | P35579 | P14618 | P68371 | P08670 |  |  |  |
| P17931 | P35579 | P43490 | Q99497 | P68371 | P08670 |  |  |  |
| P04406 | P06396 | P35579 | O96009 | P14618 | P08670 |  |  |  |
| P17931 | P35579 | O96009 | Q16378 | Q71U36 | P68371 |  |  |  |
| Q99497 | P14618 | Q71U36 | P68371 | P08670 |  |  |  |  |
| P14618 | Q16378 | Q71U36 | P68371 | P08670 |  |  |  |  |
| P22626-2 | P05787 | P98088 | P43490 | P14618 |  |  |  |  |
| P22626-2 | P17931 | P35579 | O96009 | P14618 |  |  |  |  |
| O96009 | P14618 | Q16378 | Q71U36 | P68371 |  |  |  |  |
| P62805 | P35579 | Q71U36 | P68371 | P08670 |  |  |  |  |
| P14618 | Q71U36 | P68371 | P08670 |  |  |  |  |  |
| Q99497 | P14618 | Q16378 | P08670 |  |  |  |  |  |
| O96009 | Q71U36 | P68371 | P08670 |  |  |  |  |  |
| P14618 | Q16378 | P68371 | P08670 |  |  |  |  |  |
| O96009 | Q71U36 | P68371 | P08670 |  |  |  |  |  |
| P04280 | Q16378 | Q71U36 | P68371 |  |  |  |  |  |
| O96009 | P14618 | Q16378 | P08670 |  |  |  |  |  |
| Q16378 | Q71U36 | P68371 | P08670 |  |  |  |  |  |
| P43490 | P14618 | P08670 |  |  |  |  |  |  |
| O96009 | P14618 | Q71U36 |  |  |  |  |  |  |
| P22626-2 | P17931 | P35579 |  |  |  |  |  |  |
| Q16378 | Q71U36 | P68371 |  |  |  |  |  |  |

| P14618 | P08670 |
| --- | --- |
| Q16378 | P08670 |
| Q71U36 | P08670 |
| P43490 | P14618 |
| P68371 | P08670 |
| P62805 | P22626-2 |
| P05787 | O96009 |
| P04280 | Q16378 |
| P14618 | P68371 |
| P35579 |  |
| P08670 |  |
| P68371 |  |
| P08670 |  |

| **Protein 17** | **Protein 18** | **Protein 19** | **Protein 20** | **Protein 21** | **Protein 22** | **Protein 23** |
| --- | --- | --- | --- | --- | --- | --- |
| P43490 | O96009 | Q99497 | P14618 | P04280 | Q71U36 | P68371 |
| P14618 | Q71U36 | P68371 | P08670 |  |  |  |
| Q71U36 | P68371 | P08670 |  |  |  |  |
| O96009 | P14618 | P08670 |  |  |  |  |
| P68371 | P08670 |  |  |  |  |  |
| P68371 | P08670 |  |  |  |  |  |
| Q71U36 | P08670 |  |  |  |  |  |
| P68371 | P08670 |  |  |  |  |  |
| Q71U36 | P68371 |  |  |  |  |  |
| P68371 |  |  |  |  |  |  |
| P08670 |  |  |  |  |  |  |
| P68371 |  |  |  |  |  |  |
| P08670 |  |  |  |  |  |  |
| P08670 |  |  |  |  |  |  |
